# Commentary: Pharmacological Interventions for Bacterial Prostatitis

**DOI:** 10.3389/fphar.2020.573903

**Published:** 2020-10-06

**Authors:** Vittorio Magri, Konstantinos Stamatiou, Alberto Trinchieri, Gianpaolo Perletti

**Affiliations:** ^1^ Urology Clinic, ASST Nord, Milano, Italy; ^2^ Urology Department, Tzaneio General Hospital, Piraeus, Greece; ^3^ Urology Unit, Manzoni Hospital, Lecco, Italy; ^4^ Section of Medical and Surgical Sciences, Department of Biotechnology and Life Sciences, University of Insubria, Varese, Italy; ^5^ Department of Human Structure and Repair, Ghent University, Ghent, Belgium

**Keywords:** prostatitis, chronic bacterial prostatitis, fluoroquinolones, macrolides, combination therapy

In a recent issue of this journal, Xiong and coworkers published a comprehensive article focusing on pharmacological interventions for bacterial prostatitis (Xiong et al., 2020).

After reviewing all available evidence about the etiology and current therapeutic options for both acute and chronic presentations of bacterial prostatitis, the authors provided useful diagnostic and therapeutic algorithms for both conditions.

In their algorithm, Xiong et al. include combination therapy (and in particular antibacterials combined with other antibacterials) as a possible option in case of unsatisfactory results of single-agent treatment of chronic bacterial prostatitis (CBP). According to our experience, combination of fluoroquinolones with other antibacterial agents is indeed a viable and effective therapeutic strategy, which may also hinder the emergence of resistant pathogens, in a time of dramatic increase of resistance to first-choice fluoroquinolone antibacterials (FQs).

As a *caveat* to the algorithm for CBP included in the excellent review by Xiong and coworkers, we deem important to emphasize that physicians who intend to adopt such therapeutic approach must be aware of the potential risks associated with the combination of a fluoroquinolone with a macrolide antibiotic. Since certain FQs and macrolides can cause the elongation of the QT interval of the electrocardiogram, candidate patients should be carefully interviewed and their cardiological profile evaluated before starting a course of therapy, as indicated in detail in a previous article (Magri et al., 2019). Notably, a history of long QT syndrome, or ongoing therapy with agents known to elongate the QT interval (for a list of such agents, see for instance: Tisdale, 2016) may be exclusion criteria, as addition of a FQ and/or a macrolide may increase in certain categories of patients (the elderly, for example) the risk of heart rhythm disorders (Choi et al., 2018; Gorelik et al., 2019).

Warnings considerably restricting the usage of FQs due to the possible emergence of severe adverse effects (e.g., an increased risk of neuropathy, depression, memory impairment, other neurological disorders, aneurysm, tendon rupture) have been recently published by the FDA and by the European Medicine Agency (European Medicine Agency, 2018; Food and Drug Administration, 2018). However, whereas empirical administration of FQs for *abacterial* prostatitis is no longer authorized (European Medicine Agency, 2018), FQs like ciprofloxacin or levofloxacin continue to be approved for use in patients with documented bacterial prostatitis in the USA, in the European Union and in other countries (e.g., the United Kingdom). This decision is probably due to the fact that FQs are the best available therapy for CBP in terms of antibacterial spectrum, prostatic distribution and potency, and that very few agents (trimethoprim, macrolides, carbapenems) are distributed to prostatic ducts and interstitial spaces, achieving concentrations above the MIC of Gram-negative and Gram-positive pathogens. However, since it is known that (i) pathogen eradication rates by trimethoprim are not optimal (about 60%), that (ii) macrolides do not have a broad spectrum of antibacterial activity, and (iii) that the use of carbapenems (the only beta-lactams which are sufficiently distributed to the prostate) is now restricted to the hospital setting, fluoroquinolones necessarily remain a mainstay of CBP therapy in the outpatient setting.

Given the restrictions listed above, and in the presence of worrisome fluoroquinolone resistance scenarios, research groups worldwide are investigating the efficacy of alternative antibiotics. In their review, Xiong and coworkers have summarized evidence suggesting that fosfomycin may become in the next future a safe and effective agent for treatment of CBP, especially in the presence of multidrug-resistant *Enterobacteriaceae*. Whereas fosfomycin is also available as an intravenous preparation, the vast majority of the published studies refer to the oral intake of the drug (3 grams daily, single-dose). Consensus about the optimal dosing of this agent has not been reached yet. According to our experience, a “switch protocol” similar to the one implied by Xiong et al. and adopted by Karaiskos et al. (oral fosfomycin 3 g/day for 7–10 days, switched to 3 g q48h for 6 weeks) can address at the same time the need for a “full-dosage hit” at the start of treatment, and the necessity to prevent/control diarrhea (the most common side effect of this drug) for the subsequent weeks of therapy (Karaiskos et al., 2019; Stamatiou et al., 2019). Adequately powered randomized studies are warranted to provide quality evidence concerning the effectiveness and optimal dosing of this antibiotic. Combination of fosfomycin with other antibacterial agents, possibly hindering the emergence of chemoresistance in pathogens, is highly desirable. For the moment, published evidence in this respect is missing. According to our preliminary experience, pathogen eradication and symptom remission has been achieved by combining fosfomycin with other agents, although we cannot attribute therapeutic success to the drug combination or to the prominent action of a single agent. In a series of 8 patients treated with fosfomycin and levofloxacin for 4 weeks, six patients (*E. coli*, n=4; *E. faecalis*, n=2) showed pathogen eradication and symptom resolution, whereas 2 mixed infections by *Enterococcus* and *E. coli* showed pathogen and symptom persistence. When fosfomycin was combined with beta-lactams characterized by limited prostatic penetration (co-amoxiclav, n=6; ampicillin-sulbactam, n=2), in the attempt of eradicating Gram-positive infections (*E. faecalis*, with or without concomitant *E. coli* and Staphylococci), pathogen eradication and symptom remission was achieved in all patients. As case series like this are only minimally indicative of some degree of efficacy, adequately powered studies in a randomized setting must have a final say on this issue.

According to the World Health Organization (WHO), lack of innovation in the development of new antibiotics are undermining efforts to combat drug-resistant infections, and the pipeline for antibacterial agents targeting Gram-negative pathogens appears to be particularly weak (World Health Organization, 2019). In the face of a worrisome decline of private investment in this field, public academic and non-academic research institutions represent our last hope in this regard.

## Author Contributions

GP and AT searched the literature and conceived and wrote the article. VM and KS critically appraised the literature and made an intellectual contribution to the work. All authors contributed to the article and approved the submitted version.

## Conflict of Interest

The authors declare that the research was conducted in the absence of any commercial or financial relationships that could be construed as a potential conflict of interest.
